# Supplementary material for: Synthesis of TiO2-ZnO n-n Heterojunction with Excellent Visible Light-Driven Photodegradation of Tetracycline
Source: Nanomaterials (Basel). 2024 Nov 11;14(22):1802. doi: 10.3390/nano14221802 (PMC11597633; doi:10.3390/nano14221802)
Supplement: Supplementary file 1 [file nanomaterials-14-01802-s001.zip › nanomaterials-3244643-supplementary.pdf]

nanomaterials MDPI

Supplementary Materials

Synthesis of TiO_2-ZnO n-n Heterojunction with Excellent Visible Light-Driven Photodegradation of Tetracycline

Ying Zhang *, Xinkang Bo, Tao Zhu, Wei Zhao, Yumin Cui and Jianguo Chang *

Anhui Provincial Key Laboratory of Green Carbon Chemistry, School of Chemistry and Material Engineering, Fuyang Normal University, Fuyang 236037, China; 18856643672@163.com (X.B.); zt18726384674@outlook.com (T.Z.); 13023093135@163.com (W.Z.); cymlh@126.com (Y.C.)
* Correspondence: zhangying@fynu.edu.cn (Y.Z.); jgchang@fynu.edu.cn (J.C.)

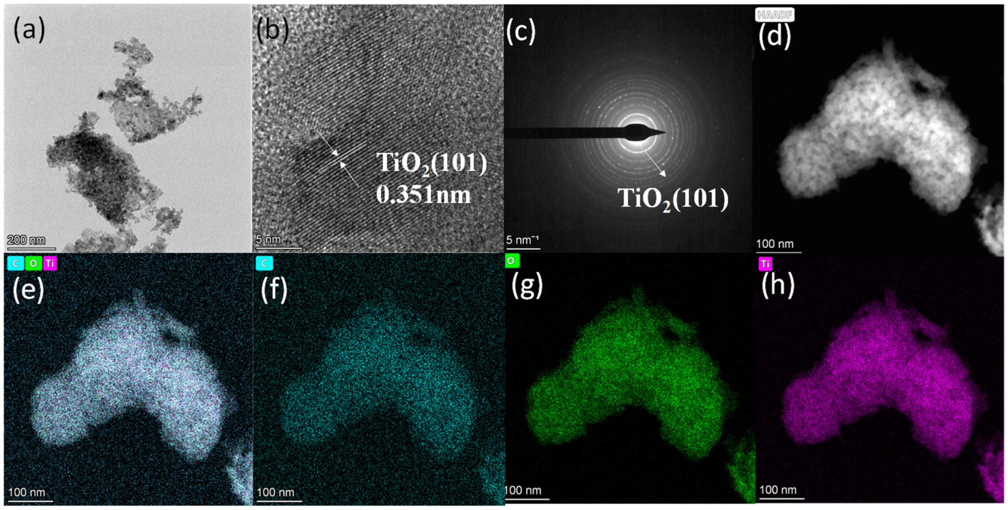

Figure S1. TEM **(a)**, HRTEM **(b,c)** and EDS mapping **(d–h)** image of the samples TiO_2.

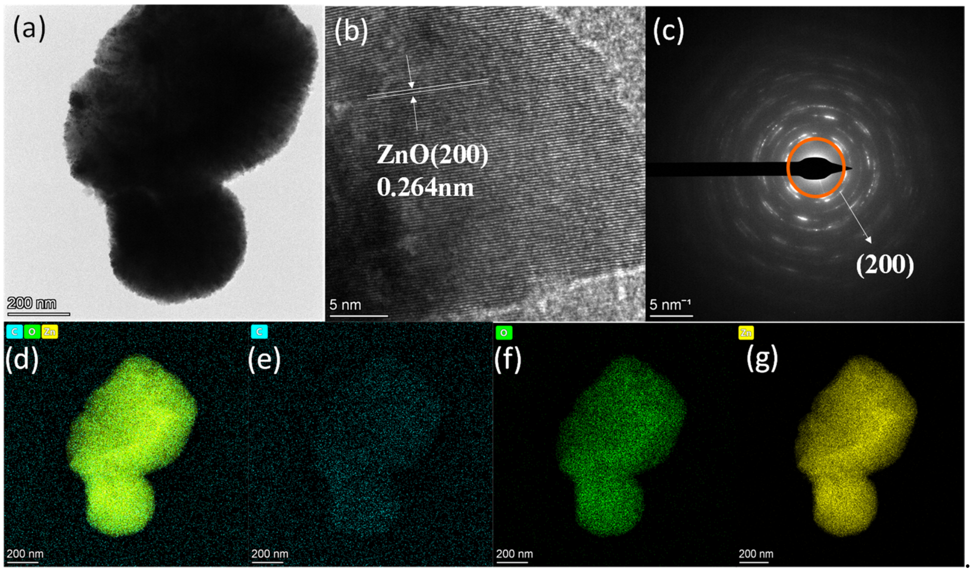

Figure S2. TEM **(a)**, HRTEM **(b,c)** and EDS mapping **(d–g)** image of the samples ZnO.

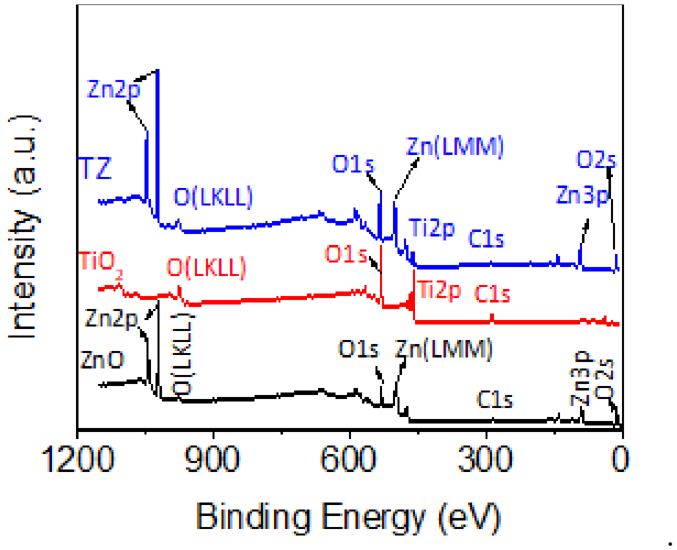

Figure S3. XPS full spectra of TiO_2, ZnO and TZ.

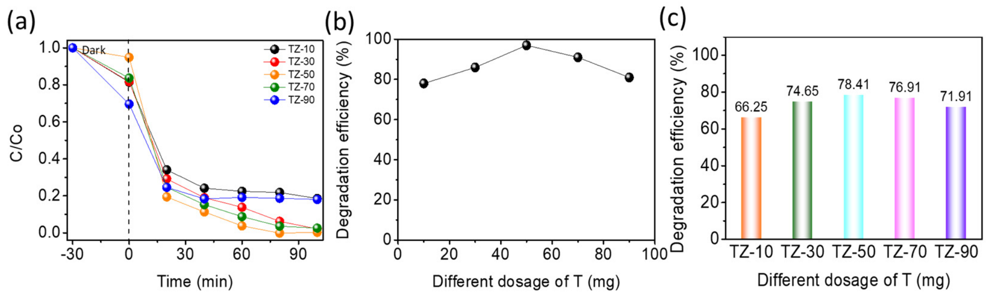

Figure S4. Photodegradation curves **(a)** and different dosage of T over TZ on the degradation efficiency **(b)** and average value **(c)** of TC under simulated sunlight.

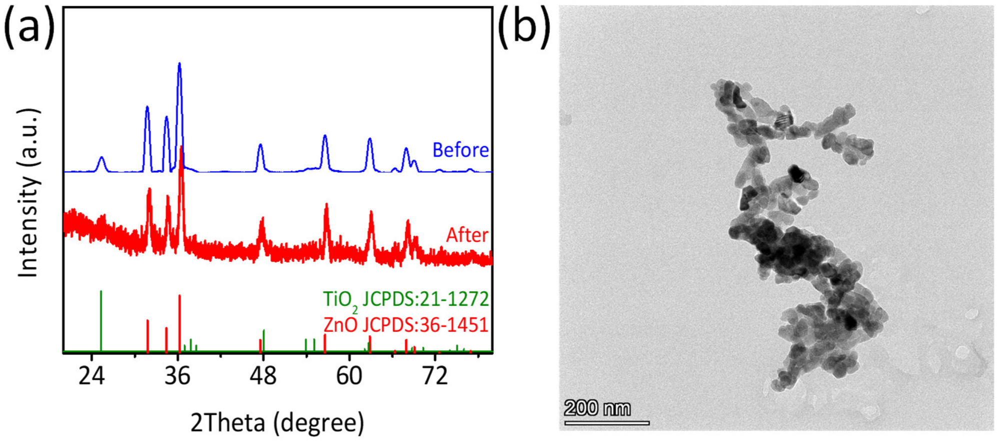

Figure S5. (a) XRD and **(b)** TEM of TiO_2-ZnO before and after four recycling runs of degradation TC.

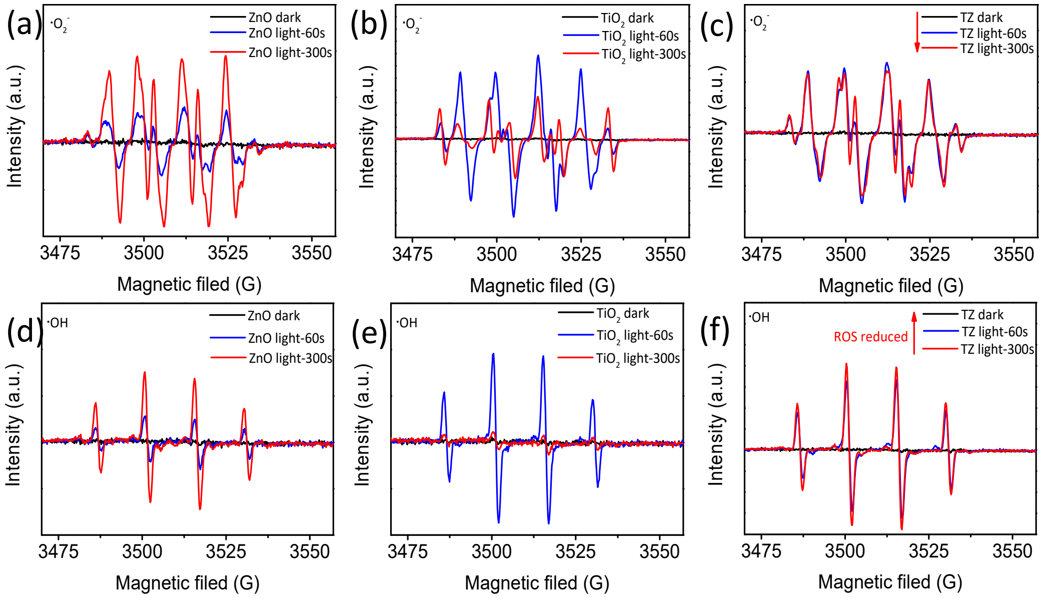

Figure S6. ESR spectra of superoxide radicals and hydroxyl radicals of ZnO **(a,d)**, TiO_2 **(b,e)** , TZ **(c,f)**.

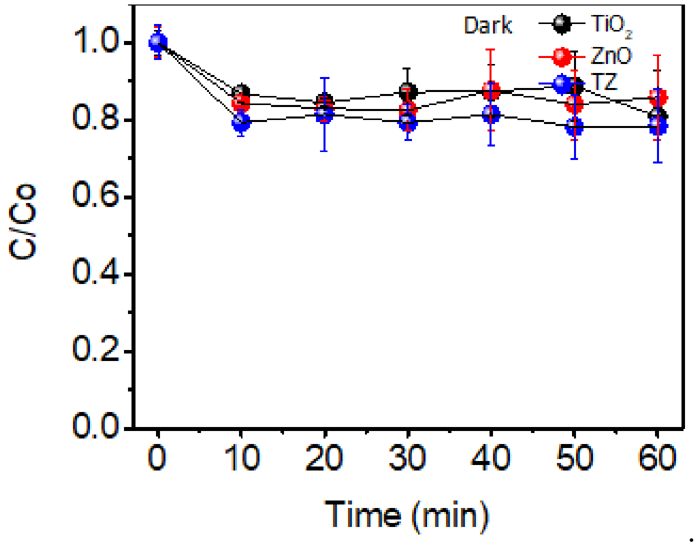

Figure S7. Adsorption studies for ZnO, TiO_2 and TZ under dark irradiation: Catalytic conditions with a catalyst concentration of 200 mg/L and a target compound (TC) concentration of 20 mg/L.
